# The burden, trends, and projections of low back pain attributable to high body mass index globally: an analysis of the global burden of disease study from 1990 to 2021 and projections to 2050

**DOI:** 10.3389/fmed.2024.1469298

**Published:** 2024-10-23

**Authors:** Chuan Zhang, Shanglin Zi, Quanzheng Chen, Shuna Zhang

**Affiliations:** ^1^Department of Physical Education and Health, Guangxi Normal University, Guilin, China; ^2^Department of Physical Education and Health, Hunan Normal University, Changsha, China

**Keywords:** high BMI, low Back pain, GBD database, trend analysis, disability-adjusted life years

## Abstract

**Objective:**

To systematically evaluate the global burden and trends of low back pain(LBP) associated with high Body Mass Index (BMI) and project future trends up to 2050 using Bayesian Age-Period-Cohort (BAPC) model, providing scientific evidence for prioritizing global preventive actions.

**Methods:**

Utilizing data from the Global Burden of Disease (GBD) 2021 study, this research analyzes the disease burden of low back pain linked to high BMI globally, with Disability-Adjusted Life Years (DALYs) as the primary metric. We examined trends by gender, age, and exposure rate using Estimated Annual Percentage Change (EAPC) and projected future trends with the BAPC model.

**Results:**

In 2021, high BMI-related low back pain accounted for 8,363,759 DALYs, with an age-standardized rate of 97.66 per 100,000 population and an EAPC of 1.14. The DALYs rate varied significantly by country, with the United States, Australia, and Eastern Europe experiencing the highest rates, all exceeding 225 per 100,000 population. The burden has increased globally, with notable rises in China, Southeast Asia, South Asia, and Africa, where EAPCs surpassed 2.5. Regions with medium and high Socio-Demographic Index (SDI) showed the most substantial increases, with the DALY rate in high SDI areas rising from 118.84 to 161.80 per 100,000, and in medium SDI areas from 41.92 to 79.10 per 100,000. Throughout the period from 1990 to 2021, females consistently experienced a higher burden of high BMI-related low back pain than males, with their DALY rate increasing from 92.01 to 126.29 per 100,000. The impact of high BMI on low back pain intensified with age, peaking in the 70–74 age group at 294.13 per 100,000, and then declining to 196.43 per 100,000 in those aged 95 and above. The BAPC model projects that by 2050, the number of DALYs will reach 15,558,278, an increase of 7,806,121 from 2021.

**Conclusion:**

From 1990 to 2021, the global burden of low back pain attributable to high BMI has intensified, particularly affecting females, younger elderly, and developed regions. With increasing global aging and obesity rates, the burden is expected to continue rising rapidly without sustained and effective targeted interventions.

## 1 Introduction

LBP is a significant global health issue, with a prevalence rate ranging from 13.1 to 20.3%. Approximately two-thirds of LBP patients transition to chronic conditions following an acute episode, leading to long-term suffering (1). The global prevalence of LBP has increased notably, with an 18% rise in incidence from 2006 to 2016. By 2020, around 619 million people were affected by LBP, and this number is projected to reach 843 million by 2050 (2, 3). As the leading cause of disability among chronic musculoskeletal disorders, LBP often results in increased medical expenses and work absenteeism, imposing a substantial economic burden. In the United States, annual expenditures on LBP range from approximately $100 billion to $200 billion, with about 150 million workdays lost due to LBP each year (4). High BMI is strongly linked to LBP, with higher BMI levels significantly increasing the risk. For every 5% increase in BMI, the risk of LBP rises by 35% (5, 6). This is due to the increased body weight associated with high BMI, which exerts greater pressure on intervertebral discs and joints, accelerating disc degeneration and the onset of intervertebral arthritis. Additionally, excessive weight burdens the muscles and ligaments surrounding the spine, exacerbating LBP symptoms (5, 7). High BMI is also associated with metabolic diseases such as type 2 diabetes, hypertension, and cardiovascular diseases, which contribute to chronic inflammation and metabolic disorders, further aggravating LBP (8). Notably, recent studies have shown that in addition to common biomechanical mechanisms, Spinal Epidural Lipomatosis (SEL) has increasingly been recognized as one of the major causes of lower back pain in patients with high BMI (9, 10). SEL occurs due to excessive accumulation of fat in the spinal canal, leading to compression of the nerve roots or dural sac, which causes spinal canal stenosis and lower back pain symptoms. This pathological mechanism has garnered increasing attention in high BMI populations (11–13).

The GBD 2021 database identifies high BMI as a risk factor for LBP. Currently, over a quarter of the global population is overweight or obese, with this trend increasing in many countries and regions. According to GBD data, from 1990 to 2021, the global adult obesity rate rose from 6.1 to 16%, and the number of obese individuals grew from 212 million to 878 million, more than a fourfold increase. Early weight management in high-risk populations can effectively reduce the risk of LBP and lower the associated prevention and control costs (14). Therefore, this study examines the global trends in the burden of LBP attributable to high BMI from 1990 to 2021, providing a foundation for the development of international LBP prevention and treatment policies and the implementation of early intervention strategies.

## 2 Data and methods

### 2.1 Study data

The data for this study is derived from the GBD 2021. This initiative, launched by the World Health Organization (WHO), the World Bank, the Bill & Melinda Gates Foundation, and various academic institutions, research organizations, and governments, aims to systematically evaluate global and regional disease burdens and their trends. The findings are intended to inform health policy and resource allocation. Since its inception in 1990, the GBD database has been actively maintained and updated with data from over 90,000 sources, including research and government publications. GBD2021 employs a standardized global methodology to estimate key metrics such as incidence, prevalence, mortality, Years of Life Lost (YLL), Years Lived with Disability (YLD), and total Disability-Adjusted Life Years (DALY) for diseases across 204 countries and regions. These estimates, which include 95% Uncertainty Intervals (UI), provide critical information for mitigating disease burdens both globally and locally.

In this study, ‘high BMI’ is defined according to the GBD database as a body mass index (BMI) exceeding the optimal health level. Specifically, GBD defines high BMI as a BMI greater than 23 kg/m^2^ for Asian populations and greater than 25 kg/m^2^ for non-Asian populations, encompassing both overweight individuals (BMI between 25 and 29.9 kg/m^2^) and those classified as obese (BMI ≥30 kg/m^2^) (3, 15). Therefore, this study included all individuals with a BMI exceeding these thresholds. We selected global DALY data attributable to high BMI for LBP from 1990 to 2021 to assess the impact of high BMI on disease burden. Additionally, since LBP does not directly lead to mortality, death data were not included in this analysis. The study also revealed that the DALY count for LBP attributable to high BMI was zero in individuals under the age of 20, and thus, data from this age group were excluded from the study.

### 2.2 Research methods

The study utilized global DALYs data from the GBD database, covering the period from 1990 to 2021. DALYs were calculated using the world standard population in the GBD database and included 95% UI to account for variability (16). As defined by Alan Lopez and Christopher Murray, DALYs are a comprehensive measure of both life quantity and quality, expressed in time units, and are widely recognized as one of the most effective indicators of global disease burden (17, 18). DALYs consist of Years of Life Lost (YLLs) due to premature death and Years Lived with Disability (YLDs), summarized by the formula: DALYs = YLLs + YLDs. The study quantified trends in age-standardized DALY rates using the EAPC. EAPC is calculated with the formula: EAPC = [exp(*β*) − 1] × 100%. An upward trend is indicated if both the EAPC value and the lower limit of its 95% Uncertainty Interval (UI) are greater than 0; conversely, a downward trend is indicated if both the EAPC value and the upper limit of its 95% UI are less than 0. If the 95% UI of EAPC includes 0, the trend is considered stable. For data analysis and visualization, the study employed Microsoft Excel 2019, Python, and R (version 4.3.2), along with packages such as ggplot2 and maps to organize and calculate age-standardized DALY rates and their EAPCs. To forecast DALY numbers from 2021 to 2050, the study also used the Bayesian Age-Period-Cohort (BAPC) and Integrated Nested Laplace Approximations (INLA) packages, ensuring accurate and reliable predictions.

## 3 Result

### 3.1 The burden of low back pain attributable to high BMI in different regions

Table 1 presents the number of DALYs, DALY rate, and EAPC from 1990 to 2021 for LBP attributable to high BMI across global regions. Overall, from 1990 to 2021, the age-standardized DALY rate increased from 70.22 (95% UI: 7.14 to 146.48) per 100,000 to 97.66 (95% UI: 9.78 to 204.00) per 100,000, with an EAPC of 1.14 (95% UI: 1.11 to 1.17). Meanwhile, the number of DALYs rose from 3,086,573 (95% UI: 312,559 to 6,484,427) in 1990 to 8,363,759 (95% UI: 840,306 to 17,424,822) in 2021. Both the number of DALYs and the DALY rate exhibited an increasing trend.

**Table 1:** DALY (disability-adjusted life years) rates, DALY numbers, EAPC (estimated annual percentage change) for LBP (low back pain) attributable to BMI in 1990 and 2021, and the trends from 1990 to 2021.

Region	DALYs (95% UI)				
	1990		2021		Eapc
	Number	Rate	Number	Rate	
Global	3086573.08 (312559.12 to 6484427.06)	70.22 (7.14 to 146.48)	8363759.33 (840306.54 to 17424821.68)	97.66 (9.78 to 204.00)	1.14(1.11 to 1.17)
Low SDI	82343.43 (9051.79 to 165429.39)	27.81 (3.09 to 55.76)	356409.63 (35002.73 to 729956.95)	50.30 (5.07 to 103.18)	1.98(1.92 to 2.04)
Low-middle SDI	276060.91 (29215.80 to 573856.68)	35.21 (3.78 to 72.71)	1227064.64 (122732.05 to 2554600.60)	71.69 (7.24 to 148.95)	2.46(2.41 to 2.52)
Middle SDI	547306.42 (57862.32 to 1153131.65)	41.92 (4.49 to 87.56)	2205381.98 (219659.90 to 4678252.07)	79.10 (7.86 to 168.24)	2.18(2.13 to 2.22)
High-middle SDI	956976.62 (96319.02 to 2014411.50)	91.90 (9.24 to 192.00)	2086188.17 (212179.44 to 4263358.45)	115.59 (11.61 to 238.00)	0.78(0.72 to 0.84)
High SDI	1218421.82 (119569.00 to 2549631.15)	118.84 (11.60 to 248.80)	2478626.07 (249211.24 to 5091840.29)	161.80 (16.00 to 332.59)	1.06(1.02 to 1.09)
Central Europe, Eastern Europe, and Central Asia	737551.28 (71920.52 to 1547896.74)	157.47 (15.31 to 329.58)	1168136.73 (117798.02 to 2408120.56)	200.95 (19.94 to 415.25)	0.85(0.81 to 0.88)
High-income	1272167.98 (124875.01 to 2651914.94)	118.15 (11.52 to 246.82)	2495269.15 (250648.79 to 5135703.96)	163.30 (16.11 to 336.14)	1.11(1.07 to 1.15)
Latin America and Caribbean	248301.86 (24318.84 to 524843.56)	87.90 (8.70 to 184.56)	905798.70 (88652.88 to 1876504.58)	141.10 (13.81 to 293.14)	1.54(1.53 to 1.56)
North Africa and Middle East	230948.98 (22552.39 to 487952.03)	104.86 (10.41 to 219.70)	1037525.34 (106622.71 to 2092695.70)	179.03 (18.31 to 358.27)	1.76(1.75 to 1.78)
South Asia	151158.96 (16625.83 to 302154.58)	19.73 (2.19 to 39.35)	796921.43 (76445.30 to 1635332.21)	46.11 (4.48 to 94.36)	3.02(2.89 to 3.15)
Southeast Asia, East Asia, and Oceania	328162.53 (39129.88 to 679715.67)	23.74 (2.85 to 48.89)	1460702.75 (149003.57 to 3067736.68)	51.75 (5.25 to 108.55)	2.87(2.73 to 3)
Sub-Saharan Africa	118281.50 (12538.05 to 240805.21)	43.46 (4.66 to 87.86)	499405.23 (49110.08 to 1041058.41)	74.89 (7.57 to 154.72)	1.74(1.71 to 1.76)
European Union	631627.83 (62174.91 to 1329178.94)	118.10 (11.56 to 248.61)	1068708.66 (104912.61 to 2178845.50)	155.72 (14.98 to 324.17)	0.89(0.86 to 0.91)
G20	2355796.05 (239141.38 to 4939134.99)	71.17 (7.24 to 148.22)	5839261.92 (588916.19 to 12089801.77)	95.80 (9.58 to 199.52)	1.04(1 to 1.07)
OECD Countries	1501972.25 (147366.40 to 3137700.62)	120.18 (11.74 to 251.29)	3078449.37 (309571.06 to 6340115.19)	165.06 (16.32 to 339.32)	1.08(1.05 to 1.1)

**Figure 1:**
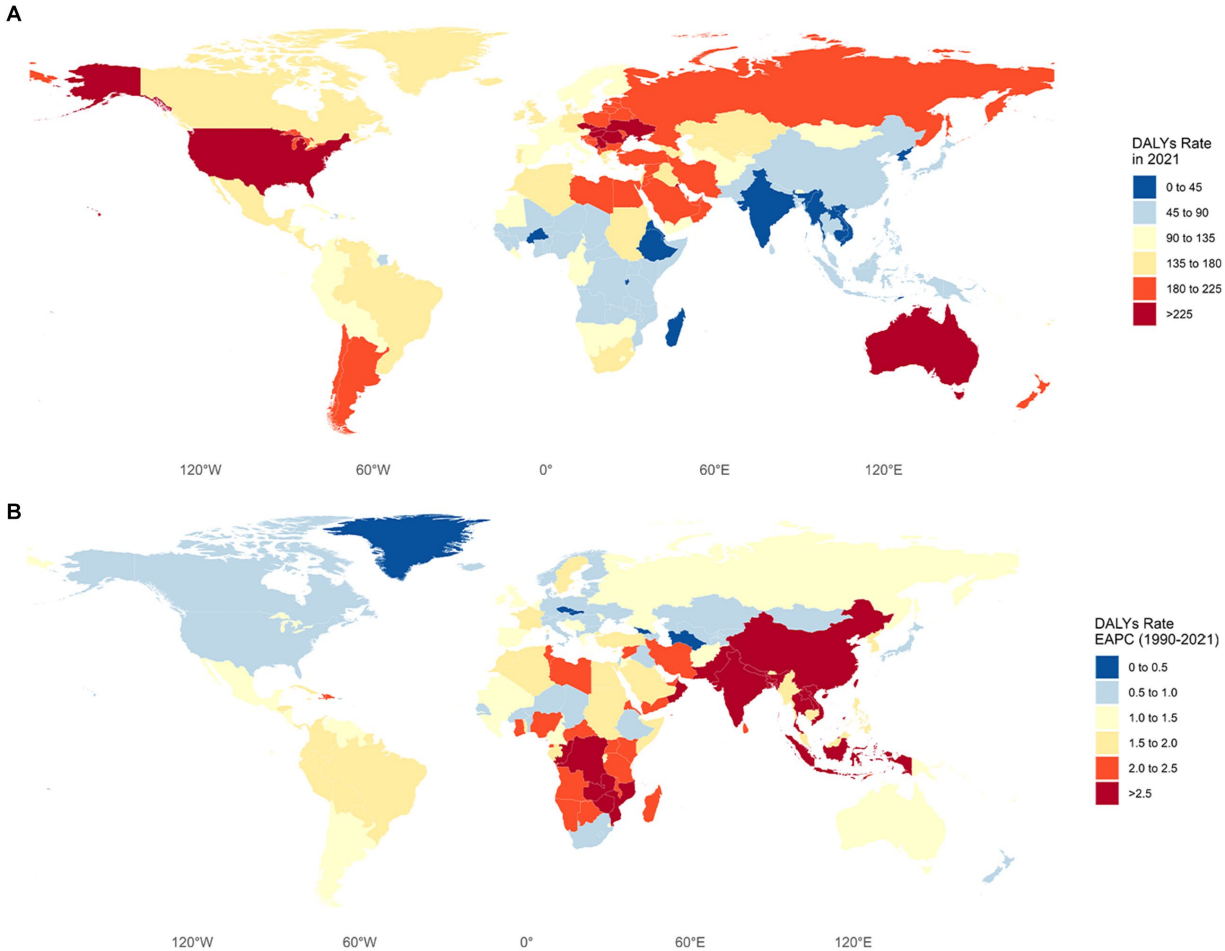
**(A)** DALY (disability-adjusted life years) rates for LBP (low back pain) attributable to high BMI in different countries and regions. **(B)** EAPC (estimated annual percentage change) for LBP (low back pain) attributable to high BMI in different countries and regions from 1990 to 2021.

Figure 1 illustrates the global distribution of DALYs and EAPC for LBP attributable to high BMI. Figure 1A reveals that the burden is particularly severe in the United States, Australia, and Eastern European countries. In the United States, the DALY rate is 240.98 per 100,000 (95% UI: 24.29–485.99), in Australia, it is 229.48 per 100,000 (95% UI: 22.85–478.53), and in Eastern Europe, countries such as Slovakia, the Czech Republic, Romania, Serbia, Montenegro, and Hungary have rates exceeding 225 per 100,000, with Hungary having the highest rate at 280.08 per 100,000 (95% UI: 27.41–576.96). Figure 1B presents the EAPC of DALY rates from 1990 to 2021, indicating a rising burden across all global regions. The increase is most pronounced in China, Southeast Asia, South Asia, and Africa, with EAPCs exceeding 2.5. In China, the EAPC is 2.89 (95% UI: 2.70 to 3.09). In Southeast Asia, Thailand exhibits the highest burden with an EAPC of 3.40 (95% UI: 3.35 to 3.74). In Africa, Zambia has the highest rate at 3.23 (95% UI: 3.15 to 3.59), and in South Asia, Pakistan shows the highest increase with an EAPC of 3.54 (95% UI: 3.35 to 3.74).

### 3.2 Trends in the burden of LBP attributable to high BMI in different SDI regions

**Figure 2:**
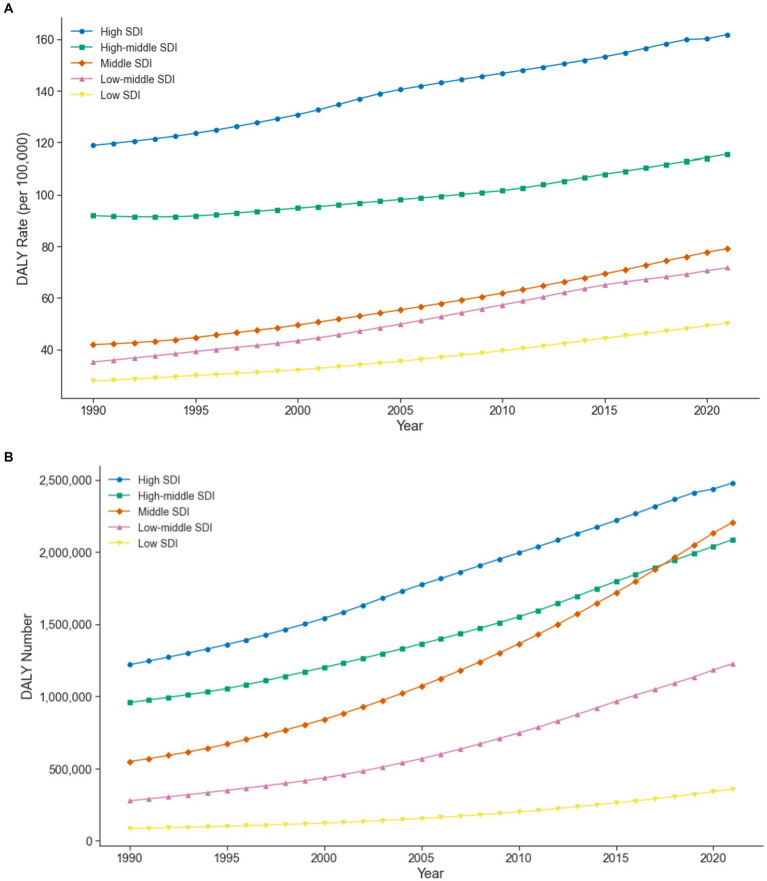
**(A)** Trends in DALY (disability-adjusted life years) rates for LBP (low back pain) attributable to high BMI in different SDI (socio-demographic index) regions. **(B)** Trends in DALY (disability-adjusted life years) numbers for LBP (low back pain) attributable to high BMI in different SDI (socio-demographic index) regions.

This study also assessed the health impacts associated with high BMI across different SDI levels. Figure 2 illustrates the DALY rates for different SDI groups. From 1990 to 2021, the DALY rate in high SDI regions increased from 118.84 per 100,000 (95% UI: 11.60–248.80) to 161.80 per 100,000 (95% UI: 16.00–332.59), show in Figure 2. In upper-middle SDI regions, it rose from 91.90 per 100,000 (95% UI: 9.24–192.00) to 115.58 per 100,000 (95% UI: 11.61–1238.00). In middle SDI regions, it grew from 41.92 per 100,000 (95% UI: 4.49–87.56) to 79.10 per 100,000 (95% UI: 7.86–168.24). In lower-middle SDI regions, it went from 21.36 per 100,000 (95% UI: 3.78–72.71) to 71.68 per 100,000 (95% UI: 7.24–148.95). In low SDI regions, it increased from 27.81 per 100,000 (95% UI: 3.09–55.76) to 50.30 per 100,000 (95% UI: 5.07–103.18). In terms of DALYs Numbers, the most significant increases were observed in high and middle SDI regions. In high SDI regions, the number of DALYs rose from 1,218,422 (95% UI: 119,569–2,549,631) in 1990 to 2,478,626 (95% UI: 249,211–5,091,840) in 2021. In middle SDI regions, the number increased from 547,306 (95% UI: 57,862–1,153,132) to 2,205,382 (95% UI: 219,660–4,678,252).

### 3.3 Trends in the burden of LBP attributable to high BMI by gender

**Figure 3:**
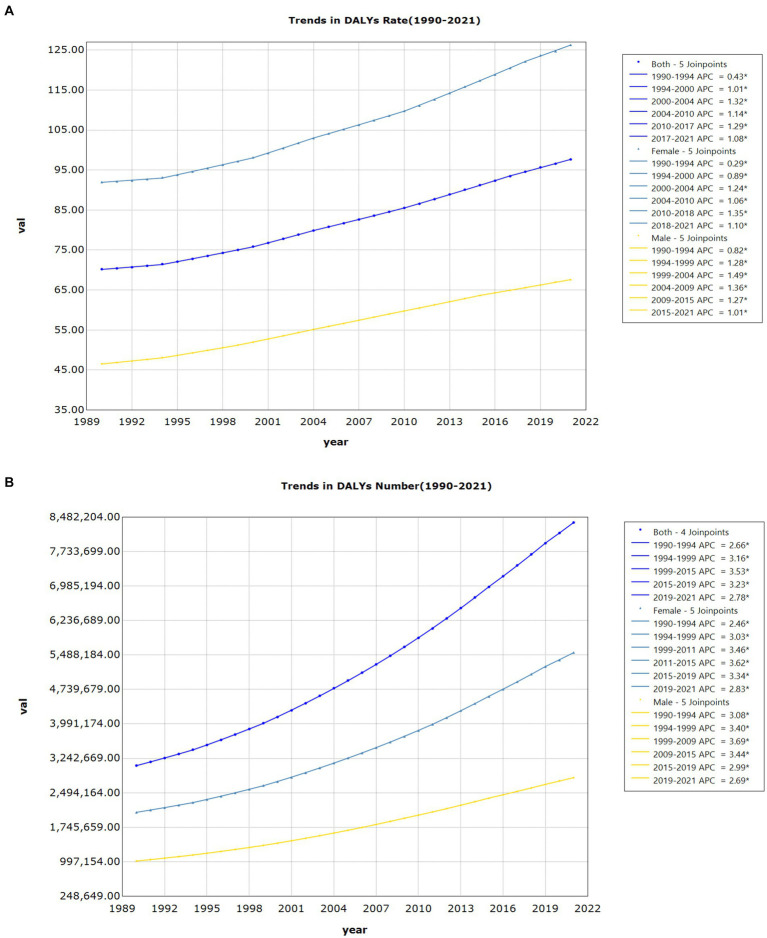
**(A)** Joinpoint regression analysis of DALY (disability-adjusted life years) rates for LBP (Low back pain) attributable to high BMI by gender. **(B)** Joinpoint regression analysis of DALY (disability-adjusted life years) numbers for LBP (low back pain) attributable to high BMI by gender.

Figure 3 illustrates the trends in DALY rates and numbers for LBP attributed to high BMI from 1990 to 2021, separated by gender. Both males and females show a continuous upward trend in these metrics. For females, the DALY rate increased from 92.01 per 100,000 (95% UI: 9.25–193.20) in 1990 to 126.29 per 100,000 (95% UI: 12.63–266.01) in 2021, while for males, it rose from 46.57 per 100,000 (95% UI: 4.84–96.25) to 67.56 per 100,000 (95% UI: 6.78–138.87). The number of DALYs for females climbed from 2,072,340 (95% UI: 207,877–4,368,723) to 5,541,374 (95% UI: 556,566–11,636,895), and for males, from 1,014,233 (95% UI: 104,683–2,115,704) to 2,822,385 (95% UI: 283,741–5,786,405).

### 3.4 Burden of low back pain attributable to high BMI across different age groups

**Figure 4:**
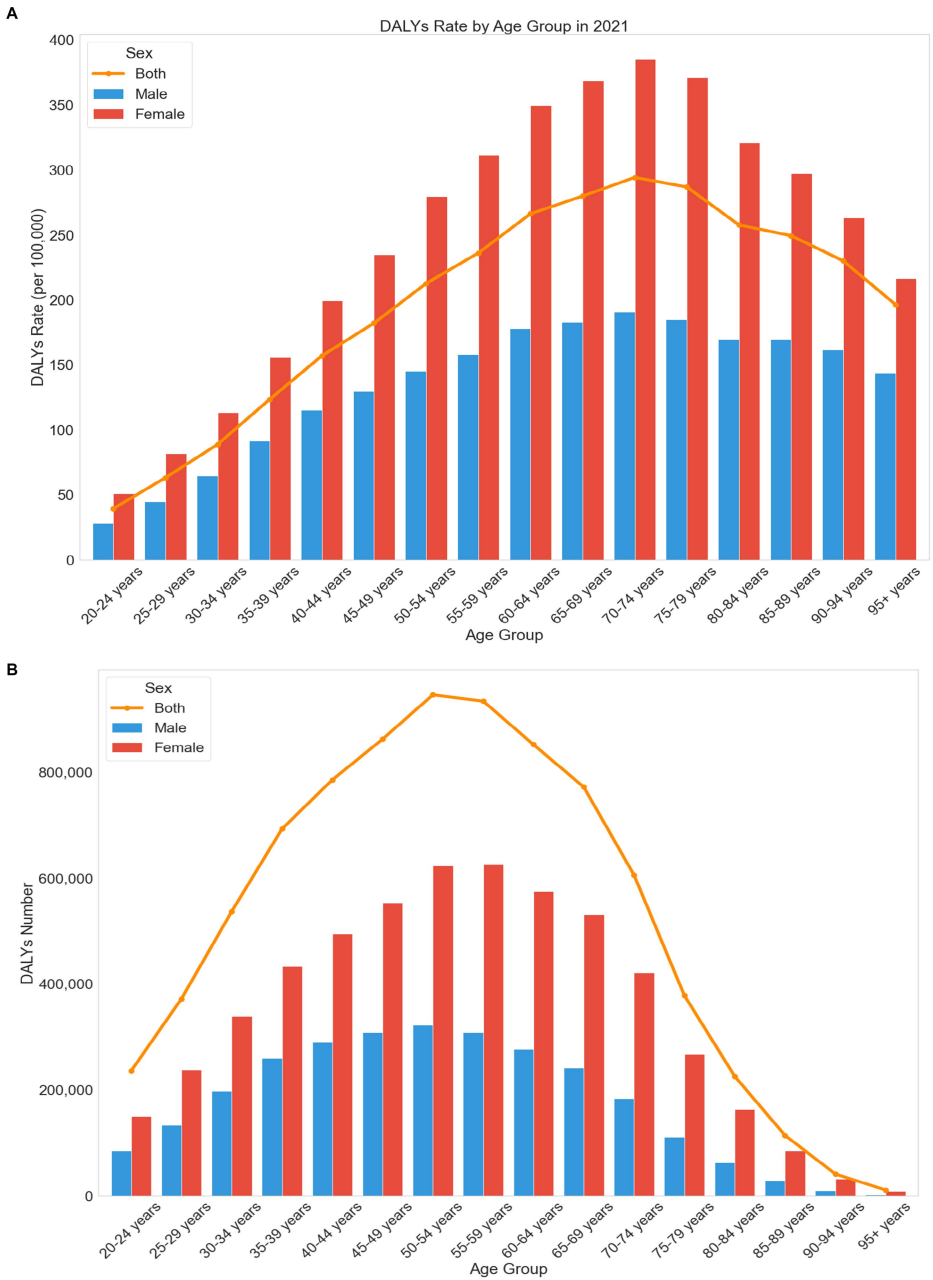
**(A)** Trends in DALY (disability-adjusted life years) rates for LBP (low back pain) attributable to high BMI across different age groups and gender. **(B)** Trends in DALY (disability-adjusted life years) numbers for LBP (low back pain) attributable to high BMI across different age groups and gender.

Figure 4 illustrates the DALY rates and numbers for LBP attributed to high BMI in different age groups and genders in 2021. The burden of LBP increases with age up to the 70–74 age group, where it peaks, and then gradually declines. Both males and females exhibit this trend. The DALY rate for the 20–24 age group is 39.46 per 100,000 (95% UI: 3.62–86.29), peaking at 294.13 per 100,000 (95% UI: 30.22–591.86) in the 70–74 age group, and decreasing to 196.43 per 100,000 (95% UI: 18.72–402.06) in the 95+ age group. Regarding the number of DALYs, the 20–24 age group has 235,630 (95% UI: 21,610–515,292), peaking at 946,039 (95% UI: 101,915–2,043,367) in the 50–54 age group, and decreasing to 10,706 (95% UI: 1,020–21,914) in the 95+ age group.

### 3.5 Projections of the burden of low back pain attributable to high BMI

**Figure 5:**
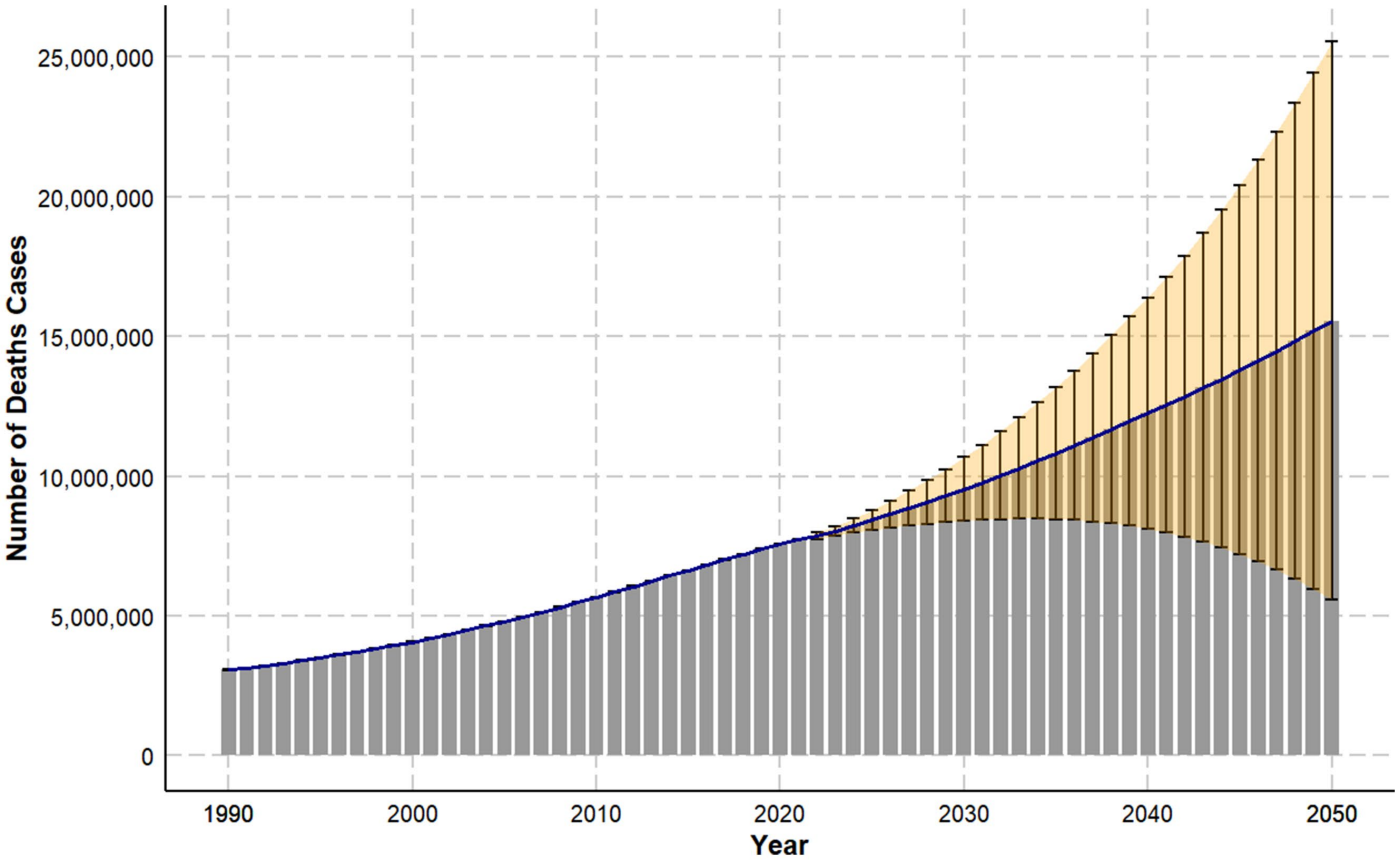
Trend projections of DALY (disability-adjusted life years) cases for LBP (low back pain) attributable to high BMI from 1990 to 2050.

Figure 5 predicts the global trend in the number of DALYs due to LBP caused by high BMI up to 2050. The overall trend indicates a continuous rise in the number of DALYs attributable to high BMI. By 2050, it is estimated that the number of DALYs will reach approximately 15,558,278 (95% UI: 5,577,584–25,538,972), representing an increase of 7,806,121 from 2021, with a growth rate of about 200.70%. Compared to 1990, this represents an increase of approximately 12,471,705, with a growth rate of around 504.06%.

## 4 Discussion

This study is the first to systematically analyze the global burden of LBP attributable to high BMI from 1990 to 2021 and predict future trends. Overall, in 2021, the number of DALYs due to high BMI-related LBP increased by approximately 5.28 million compared to 1990, with the DALY rate increasing by about 27.39 per 100,000 people, and an EAPC of 1.14 (1.11 to 1.17). Both the number and rate showed significant increases. This phenomenon can primarily be attributed to the sharp rise in global obesity rates. According to WHO data, the global adult obesity rate was 6.1% in 1990, with approximately 212 million obese adults. By 2021, the obesity rate had risen to 16%, with the number of obese adults reaching about 878 million. The obesity rate nearly tripled, and the number of obese individuals more than quadrupled (14). This trend is mainly due to lifestyle changes, including the shift towards high-calorie, high-fat, and high-sugar diets, and a significant reduction in physical activity (19). The acceleration of urbanization has further promoted a sedentary lifestyle, leading not only to weight gain but also to decreased physical activity levels, thereby increasing the risk of obesity-related health problems (20). Additionally, population aging is another crucial factor. According to WHO statistics, the global population aged 60 and above was about 380 million in 1990, rising to approximately 1.04 billion by 2020, a 2.7-fold increase (14). The increase in the elderly population means more people are at high risk for chronic diseases, and the relationship between high BMI and LBP is particularly significant among the elderly. Older adults are more susceptible to weight-related issues due to joint degeneration and osteoporosis, leading to an increased incidence of LBP. This underscores the urgent need to improve chronic disease management systems (21, 22).

It is particularly noteworthy that recent studies have revealed a significant factor that may have been overlooked in previous research. In recent years, SEL has been widely recognized as one of the primary causes of lower back pain in patients with high BMI, especially in those who are overweight or obese (10). The pathological mechanism of SEL mainly involves excessive fat accumulation in the spinal canal, leading to compression of the nerve roots or dural sac, which in turn causes spinal canal stenosis, lower back pain, and other spinal disorders (11–13). Among obese patients, MRI studies have shown that the prevalence of SEL can range from 15 to 24% (11, 13). However, due to the clinical presentation of SEL being similar to other causes of lower back pain, such as intervertebral disc degeneration or arthritis, along with insufficient imaging assessments and lack of awareness about SEL, its diagnostic rate is significantly underestimated, and the actual incidence may be much higher than currently reported data (13). Further research has demonstrated that for every unit increase in BMI, the volume of epidural fat increases by approximately 45 mm^3^, significantly raising the risk of SEL (12). As fat accumulation and compression intensify, the burden on the spine increases, further exacerbating LBP symptoms. Moreover, weight loss surgery has shown a significant effect in alleviating SEL symptoms. In obese patients, the volume of epidural fat significantly decreased from 82 ± 25.5 mm^3^ before surgery to 46 ± 20 mm^3^ after surgery, representing about a 40% reduction. Following weight reduction, patients experienced significant improvement in neurogenic claudication and lower back pain symptoms, further confirming the impact of weight on SEL and LBP (9, 10). Therefore, although the GBD database does not provide specific data on SEL, existing clinical evidence suggests that SEL, as an important pathological mechanism linking high BMI and lower back pain, is greatly underestimated and warrants more attention in future clinical diagnoses and research. In practice, there is an urgent need to enhance imaging screening and diagnostic awareness of SEL, particularly in patients unresponsive to conventional treatments, to improve the diagnosis rate and prognosis of SEL.

Regional differences show significant variation in the burden of LBP caused by high BMI. The most severely affected areas include the United States, Australia, and certain Eastern European countries. Despite being developed regions with advanced medical technology, the prevalence of fast food culture in these countries leads to high consumption of high-fat, high-sugar, and high-salt foods, greatly exacerbating obesity issues (19). Moreover, sedentary work environments prevalent in these areas, characterized by long hours sitting in front of computers, further increase the risks associated with high BMI and LBP (16, 23, 24). In contrast, the regions with the highest growth rates in LBP burden due to high BMI are developing countries such as China, Southeast Asia, South Asia, and Africa. China’s EAPC reached 2.89, while Thailand in Southeast Asia and Zambia in Africa reached 3.40 and 3.23, respectively. Rapid economic development and urbanization in these regions have aggravated health problems related to high BMI. For instance, in China, advancements in technology have altered labor structures, leading to more sedentary lifestyles and reduced physical activity, thereby increasing the risks of high BMI and LBP (18, 25, 26). To address these issues, developed countries can implement measures to improve dietary habits and increase physical activity. Strategies include imposing higher taxes on high-fat, high-sugar, and high-salt foods, enhancing the availability of healthy foods, promoting the use of standing desks, and encouraging regular physical activity. For developing countries, prioritizing the development of public infrastructure and health education is crucial. Improving urban planning, providing more public fitness facilities and activity spaces, and promoting walking and cycling can significantly reduce the burden of LBP related to high BMI.

The burden of LBP caused by high BMI is significantly higher in females than in males. From 1990 to 2021, the DALY rate for females increased from 92.01 per 100,000 to 126.29, while for males it rose from 46.57 to 67.56. This disparity is largely due to the loss of estrogen protection in postmenopausal women, which decreases basal metabolic rate and makes weight gain and abdominal obesity more likely, thus increasing BMI. Flegal et al. found that the obesity rate among women over 65 is about 5% higher than in men, which further elevates the risks of insulin resistance and type 2 diabetes, leading to a higher disease burden (27). Additionally, reduced estrogen levels contribute to bone loss and decreased muscle mass and strength, making women more susceptible to low back pain, intervertebral disc herniation, and other related issues, thus hindering their physical activity (28). To address these challenges, countries should implement social policies and public health measures aimed at increasing women’s participation in physical activities and improving their health. Such measures could include expanding access to sports facilities, enhancing health education and awareness, prioritizing women’s health issues, and promoting gender equality. These actions will help mitigate the negative effects of low physical activity on women’s health and foster societal health development and gender equality.

In addition, age differences significantly impact the burden of LBP caused by high BMI. Before age 70, the burden of LBP due to high BMI increases with age, peaking at 70–74 years old, and then gradually decreases. The DALY rate for the 20–24 age group is 39.46 per 100,000, reaching its highest at 294.13 per 100,000 for the 70–74 age group, and decreasing to 196.43 per 100,000 for those aged 95 and above (95% UI: 18.72–402.06). This trend is attributed to the gradual weakening of muscle strength with age. According to Nilwik et al., muscle strength decreases by about 1 to 2% per year starting at age 40, increasing to 1.5 to 3% per year by age 60, along with a concurrent annual loss of about 1 to 2% of motor neurons. Additionally, people over 60 have about 30 to 40% fewer type II muscle fibers compared to younger individuals, and type II muscle fibers play a crucial role in maintaining muscle strength (29, 30). Furthermore, the basal metabolic rate decreases with age, with an average decline of 5 to 10% per decade after age 50 (31, 32). These physiological changes make the elderly more susceptible to the effects of high BMI, increasing the incidence of low back pain. Therefore, countries should focus more on middle-aged and elderly populations by increasing social support and developing specific exercise prescriptions. This requires joint efforts from the scientific community and society to help the middle-aged and elderly maintain health. The decrease in burden after age 70 is primarily due to the significant increase in mortality rates. For example, the annual mortality rate is about 25% for those aged 75–79, about 30% for those aged 80–84, and over 35% for those aged 85 and above. Additionally, the proportions of individuals over 70 using pain medications and physical therapy are 50 and 40%, respectively, significantly higher than the 30 and 20% in those under 70. People over 70 are more attentive to health management, with 30% regularly participating in exercise, significantly higher than the 20% in those under 70. These factors collectively explain the decrease in the burden of LBP after age 70 (33, 34).

This study has several limitations. First, it primarily relies on the GBD database. While the GBD database offers extensive data on disease burden, differences in data collection methods and standards across countries and regions might affect the reliability of the results. Second, this study does not thoroughly explore the specific implementation effects and mechanisms of different policies. Future research should focus on analyzing the specific effects of policy implementations in various countries and regions to provide scientific evidence for policy-making.

## 5 Conclusion

This study is the first to highlight the global burden of low back pain caused by high BMI. The findings indicate that both the number and rate of DALYs due to high BMI-related low back pain have continuously increased, with projections showing significant further increases by 2050. Regionally, high SDI and middle SDI areas bear the greatest burden and show the largest increases. The impact on females is higher than on males, and this gap is widening. Additionally, the burden of low back pain caused by high BMI increases with age, peaking at 70–74 years, and then gradually decreases after age 70.

## Data Availability

The original contributions presented in the study are included in the article/supplementary material, further inquiries can be directed to the corresponding author.
